# Democratising artificial intelligence in healthcare: community-driven approaches for ethical solutions

**DOI:** 10.1016/j.fhj.2024.100165

**Published:** 2024-09-19

**Authors:** Ceilidh Welsh, Susana Román García, Gillian C. Barnett, Raj Jena

**Affiliations:** a Department of Oncology, University of Cambridge, Cambridge, UK; b Centre for Discovery Brain Sciences, College of Medicine & Veterinary Medicine, Biomedical Sciences, University of Edinburgh, UK; c Addenbrookes Hospital, Cambridge University Hospitals, Hills Road, Cambridge, UK

**Keywords:** Artificial intelligence, Data ethics, Healthcare, Open science, Patient engagement, Collaboration

## Abstract

The rapid advancement and widespread adoption of artificial intelligence (AI) has ushered in a new era of possibilities in healthcare, ranging from clinical task automation to disease detection. AI algorithms have the potential to analyse medical data, enhance diagnostic accuracy, personalise treatment plans and predict patient outcomes among other possibilities. With a surge in AI's popularity, its developments are outpacing policy and regulatory frameworks, leading to concerns about ethical considerations and collaborative development. Healthcare faces its own ethical challenges, including biased datasets, under-representation and inequitable access to resources, all contributing to mistrust in medical systems. To address these issues in the context of AI healthcare solutions and prevent perpetuating existing inequities, it is crucial to involve communities and stakeholders in the AI lifecycle.

This article discusses four community-driven approaches for co-developing ethical AI healthcare solutions, including understanding and prioritising needs, defining a shared language, promoting mutual learning and co-creation, and democratising AI. These approaches emphasise bottom-up decision-making to reflect and centre impacted communities' needs and values. These collaborative approaches provide actionable considerations for creating equitable AI solutions in healthcare, fostering a more just and effective healthcare system that serves patient and community needs.

## Introduction

The meaning of artificial intelligence (AI) can be interpreted in many ways, depending on the context and individuals involved.^1^^,^^2^ Therefore, before exploring the complexities of ethical AI in healthcare, it's important to establish common ground with a definition of AI for this discussion. Here, AI is defined as a machine's ability to display ‘human-like’ capabilities such as reasoning, learning, planning and creativity; enabling computer systems to process large quantities of data, learn from patterns, and solve problems to support healthcare providers. The rapid advancement and widespread adoption of AI has ushered in a new era of possibilities in healthcare, ranging from clinical task automation to disease detection.^3^, ^4^, ^5^ AI algorithms have the potential to analyse medical data, enhance diagnostic accuracy, personalise treatment plans and predict patient outcomes, among other possibilities.

However, with the surge in AI's popularity, its developments are outpacing policy and regulation^4^^,^^5^ and solutions are predominantly developed within closed silos.^6^^,^^7^ This raises concerns about whether AI solutions are being developed collaboratively and ethically, with proactive risk mitigation, or whether there is a growing reliance on reactive risk management post-deployment, after unintended consequences have already had their impact.^7^

While ethical principles and risk management systems are already a vital part of healthcare, from data privacy standards to research ethics committees and third-party regulatory bodies,^8^^,^^9^ healthcare is not without its own ethical issues. A legacy of biased datasets, under-representation, lack of informed consent in clinical trials, and inequitable access to healthcare resources all contribute towards mistrust in medical systems.^10^^,^^11^ Without centring communities in the design, development and deployment of AI solutions, there is a risk of cementing this mistrust and furthering existing ethical issues. Therefore, a crucial question emerges. How do we create ethical AI solutions that improve patient outcomes and experiences, without perpetuating existing risks and introducing new ones?

To start exploring the topic of collaboration for ethical AI, small-group and one-to-one discussions were held with patients, healthcare professionals and researchers. From these discussions, four key approaches emerged:

• Understanding and prioritising need
• Defining shared language
• Mutual learning and continued co-creation
• AI democratisation

These community-driven approaches draw inspiration from grassroots movements, prioritising bottom-up decision-making and reflecting the needs of the communities impacted by AI, rather than top-down directives. It is important to note that, due to this articleʼs scope and the nature of small-group discussions, the following topics represent a limited number of people, including the authors, and a small subset of communities and stakeholders (see acknowledgements). Discussions with these individuals have been instrumental for reflecting on the role of these four proposed approaches on ethical AI in healthcare. Their contributions highlight how we can foster the collaborative conditions necessary for continuously involving communities and stakeholders in the AI lifecycle. These approaches present actionable considerations to build upon, and co-create with the current research on community-driven and ethical AI.^12^, ^13^, ^14^, ^15^

## Understanding and prioritising need

Developing an ethical AI solution requires understanding and prioritising the diverse needs of all involved communities and stakeholders. Without collaboration, critical questions regarding patient and healthcare system needs, and the suitability of AI to meet these needs, may remain unanswered. To collaboratively develop an ethical solution, it is vital to identify, engage and prioritise community and stakeholder needs.

**Fig. 1:**
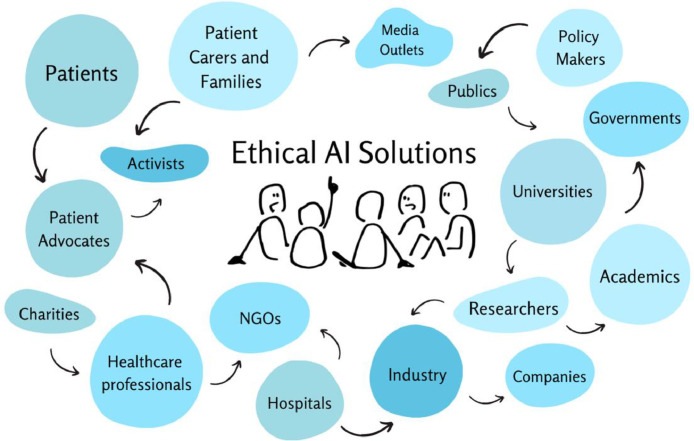
A map of communities and stakeholders involved in the AI lifecycle. Communities are groups directly affected or impacted by AI solutions, and stakeholders are those with direct investment in, or control over, AI development, implementation, and governance. Varying shapes and colours indicate that engagement and co-creation happens in different ways, but do not indicate hierarchical importance. The multi-directional arrows show the interconnectedness across all groups for co-developing ethical AI solutions. (For interpretation of the references to colour in this figure legend, the reader is referred to the web version of this article.)

*Identify* the communities and stakeholders involved in the AI solutions lifecycle. When we discuss communities, it is vital to understand who and where they are, which can be challenging as they are not always homogenous, visible or accessible. Identifying who is involved or impacted requires outlining the AI solution's purpose, scope and impact. This allows reflection on the solution's cultural and geographical context; diversity, representation, and inclusion; and existing or potential relationships, networks or partnerships. In healthcare, this can look like a complex web with many interactions and impacts (Fig. 1).

*Engage* with identified communities and stakeholders to holistically understand the problem which the AI solution aims to address. This engagement must be continuous, consistent and iterative from before design to post-deployment evaluation of the solution to ensure that patient and healthcare system needs are addressed. This requires ongoing commitment, effort and resources to support all communities and maintain equitable access to the collaborative process.

*Prioritise* patient perspectives, continuous collaboration and the integration of communities and stakeholders. Typically, healthcare solutions often consider clinical effectiveness, cost-effectiveness and patient requirements, supported by clinical and economic research.^16^ However, they seldom prioritise patient perspectives and experiences, or the capacity of current healthcare infrastructures. Patient and Public Involvement and Engagement (PPIE) networks are one example of prioritising experiences and perspectives when developing healthcare solutions. There is no single approach to PPIE, and many organisations have established their own frameworks for PPIE across different research domains.^17^, ^18^, ^19^ However, it is crucial that PPIE is prioritised from the outset and viewed as an ongoing process of co-creation, with the understanding that patient and public engagement is not a tick-box exercise. Prioritising continuous collaboration and integration of community and stakeholder feedback throughout the AI lifecycle puts community needs and user experiences at the heart of the solution.

## Defining shared language

In addition to identifying, engaging and prioritising communities and stakeholders, it is important to ensure mutual understanding of patient and healthcare system needs. Language is crucial for sharing understandings, communicating values and, in medical contexts, shaping therapeutic relationships.^20^ Any complex web of collaborations will encounter varying language due to technical terms or jargon, culture-specific language, regional, national and social differences and interpretations.^21^

**Fig. 2:**
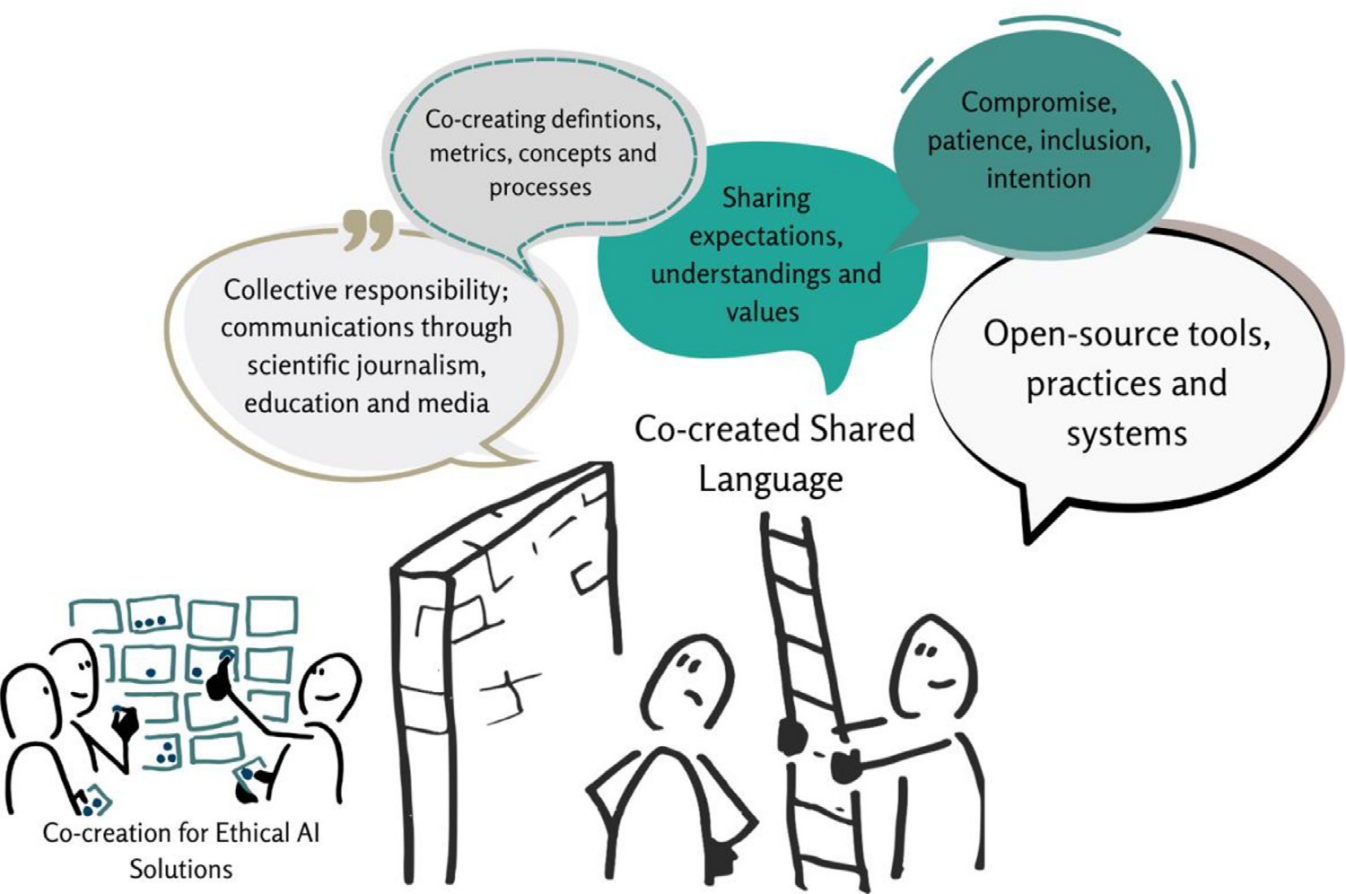
Some of the suggested components for co-creating a shared language between communities and stakeholders. These different approaches seek to overcome barriers to understanding the range of patient and healthcare system needs across the many groups involved.

Co-creating shared language can happen on multiple levels (Fig. 2). Interpersonal co-development can include citizen juries, open forums, patient and public engagement and stakeholder focus groups. Representation of all communities and stakeholders is important to highlight differences in definitions, terms and concepts, and begin the process of collectively defining a shared vocabulary. This collaborative approach enhances communication by avoiding assumptions, enabling clearer goals and reducing ambiguity and jargon.

More broadly, open-access tools, practices and systems can establish comprehensive shared language that lays the foundation for engaging in conversations that co-create domain-specific vocabularies. Work such as the Data Hazards Project^22^ presents one example of how community-driven tools can develop an interdisciplinary vocabulary for discussing and reflecting on the ethical risks associated with data science and AI. Vitally, the importance of this shared language stretches beyond our interpersonal collaborations and open-source tools, and into the collective responsibility for how AI is discussed in scientific journalism, education systems and in media representation.

## Mutual learning and continued co-creation

Shared language is just one aspect in a vast and intricate ecosystem of co-creation. AI co-creation aims to establish sustainable, collaborative partnerships focused on mutual learning and knowledge exchange for the design and development of ethical AI solutions. This approach means that patients and patient carers are no longer recipients of a service, and healthcare providers are no longer end-users of an AI tool, but are the active co-creators, sharing their knowledge and experiences. This collaborative effort can dispel AI myths and misconceptions, promote ethical use of AI, and generate best practice guidelines for the development of regulatory policies.^23^, ^24^, ^25^

Different communities and stakeholders are already co-creators in AI solutions, although they often go unrecognised. For example, patients contribute to AI through their consented medical data. These data are essential for developing, training and validating AI models, and for creating inclusive, representative and ethical training datasets. Since patient data are central to the generation of AI systems, actively co-creating with patients' experiences, perspectives and input into ethical AI development should start at the solution's ideation. Therefore, mutual learning continues to be fostered as the AI solution is created, identifying biases, privacy concerns and ethical challenges early in development.

Supporting mutual learning and co-creation throughout the AI lifecycle requires a ‘sandwich’ approach.^26^ This approach combines bottom-up processes, like community-driven design and co-created shared language, with top-down policies and incentives. Regulatory frameworks are essential for assessing AI before and after deployment to help mitigate risks and keep communities and end-users central to the solution. These frameworks and policies must represent diverse stakeholders, be anticipatory and challenge any current systems that drive or incentivise research through profit alone.

Co-creation can also involve designing AI systems with human-in-the-loop approaches, where these systems support, rather than replace, clinical decision-making and workflows.^27^ These incorporation of human interaction ensures that human agency is required for critical decision-making steps.^28^ Interactions may include training systems to perform a specific task correctly, validating and verifying results for edge cases, identifying counterintuitive results or biases, and providing continuous feedback to update system performance. By integrating human intelligence, judgement and interaction into the loop, both trust and transparency in the system's decision-making process are improved.^25^^,^^29^

## AI democratisation

AI democratisation represents a paradigm shift for innovation, contribution and regulation.^30^ It aims to decentralise power so that AI and its perceived benefits are accessible to a wider range of people, organisations and communities, regardless of technical expertise or resources.^31^

Democratisation of design and development is shaped by co-creation, participatory design and mutual learning. Each community, stakeholder and individual involved in this process brings unique perspectives shaped by their identity, influence and experiences, known as positionality.^32^ Different positionalities can introduce power dynamics and biases between groups, and their interactions with the AI solution. Part of democratisation through co-creation requires evaluating these positionalities, recognising and challenging any resulting risks, to promote more inclusive and equitable decision-making. Reflective and co-creation processes provide the space to reflect on when not to design, to challenge AI technological solutionism,^33^ and to reflect on the roots of current co-design discourse and who this discourse predominantly serves.^34^

Technological democratisation requires implementing open-source, reproducible systems, and decentralised AI model training and testing. Federated learning,^35^ for example, can democratise AI model design by training across institutions without sharing sensitive patient or health data.^36^ This approach aims to mitigate algorithmic bias by diversifying institutions and patient demographics included in the training and testing datasets. It also creates infrastructure for cross-border and cross-institution model validation. Democratised AI development could involve mandatory reporting and auditing, such as an AI model ‘passport’ to trace AI development before implementation.^37^ These ‘passports’ evidence the use of version controlling, stress-testing, availability of open-access code for review, the use of anonymised shared data or synthetic data for external testing, and evidence of validation studies.

As with other medical interventions, it is crucial to conduct rigorous patient trials to ensure that AI systems are safe and regulated before they are integrated into clinical practice. Additionally, it is vital to ensure that AI systems have the supporting security infrastructure to protect the increasing quantities of patient data. Ensuring that patient trials and security infrastructure are implemented in the AI lifecycle requires democratised development of new policies and governance frameworks.

Democratising AI policy and governance requires processes to facilitate the representation of diverse and often conflicting beliefs, opinions and values into decisions about how AI is governed. Policy should incentivise co-creation outside the developer echo-chamber to mitigate risks and broaden perspectives. Policy and governance should reward time as part of grants for collaborative co-creation, incentivising and investing funding into collaborative and community-driven projects.

## Conclusion

AI implementation in healthcare has the potential to improve patient outcomes and streamline clinical workflows for enhanced healthcare experiences. This piece discusses how current AI lifecycle processes often exclude those impacted by proposed solutions, and discusses several community-driven approaches for building collaborative and ethical AI solutions. These approaches focus on understanding community and stakeholder needs, co-developing a shared language, co-creating from diverse perspectives, and democratising control over AI design, development and deployment. These present some actionable starting points for creating ethically empowered AI in healthcare that centres patients, community collaboration and stakeholder involvement for a more just and equitable healthcare system.

## Funding

Ceilidh Welsh, Gillian C. Barnett and Raj Jena are funded by Cancer Research UK RadNet Cambridge [C17918/A28870]. Susana Román García is funded by Medical Research Scotland and CERN openlab (PHD-50053-2019). Ceilidh Welsh and Susana Román García were supported by the Alan Turing Institute's Enrichment Scheme.

## CRediT authorship contribution statement

**Ceilidh Welsh:** Writing – review & editing, Writing – original draft, Investigation, Conceptualization. **Susana Román García:** Writing – review & editing, Conceptualization. **Gillian C. Barnett:** Writing – review & editing. **Raj Jena:** Writing – review & editing, Conceptualization.

## Declaration of competing interest

The authors declare that they have no known competing financial interests or personal relationships that could have appeared to influence the work reported in this paper.
